# Asian American Representation in Medicine by Career Stage and Residency Specialty

**DOI:** 10.1001/jamanetworkopen.2024.44478

**Published:** 2024-11-19

**Authors:** Patricia Mae G. Santos, Carlos Irwin A. Oronce, Kanan Shah, Fumiko Chino, Mylin A. Torres, Reshma Jagsi, Curtiland Deville, Neha Vapiwala

**Affiliations:** 1 Department of Radiation Oncology, Winship Cancer Institute, Emory University, Atlanta, Georgia; 2 Department of Medicine, VA Greater Los Angeles Healthcare System, Los Angeles, California; 3 Division of General Internal Medicine and Health Services Research, University of California Los Angeles; 4 Department of Internal Medicine, New York University Grossman School of Medicine, New York; 5 Department of Radiation Oncology, MD Anderson Cancer Center, Houston, Texas; 6 Department of Radiation Oncology and Molecular Radiation Sciences, Johns Hopkins University School of Medicine, Baltimore, Maryland; 7 Department of Radiation Oncology, Perelman School of Medicine at the University of Pennsylvania, Philadelphia

## Abstract

**IMPORTANCE:**

Asian American individuals are not underrepresented in medicine; however, aggregation in prior workforce analyses may mask underlying disparities.

**OBJECTIVE:**

To assess representation by Asian race and disaggregated subgroups in the US allopathic medical school workforce.

**Design, Setting, and Participants:**

This cross-sectional study used Association of American Medical Colleges (AAMC) special reports, generated using the AAMC Applicant-Matriculant Data File, Student Records System, Graduate Medical Education Track Survey, and faculty roster. Participants included medical school applicants, matriculants, graduates, residents, and faculty enrolled or employed at US allopathic medical schools between 2013 and 2021. Data were analyzed between March and May 2024.

**EXPOSURE:**

Asian race or ethnic subgroup as per AAMC and US Census Bureau Office of Management and Budget criteria, including Bangladeshi American, Cambodian American, Chinese American, Filipino American, Indian American, Indonesian American, Japanese American, Korean American, Pakistani American, Taiwanese American, and Vietnamese American.

**MAIN OUTCOMES AND MEASURES:**

Representation quotients (RQ) were used to indicate representation that was equivalent (RQ of 1), higher (RQ greater than 1), or lower (RQ less than 1) than expected representation based on US population estimates. One-way analysis of variance and linear regression models assessed mean RQ differences by career stage and over time, with Bonferroni correction for multiple comparisons.

**RESULTS:**

In this study, Asian American individuals accounted for 94 934 of 385 775 applicants (23%), 39 849 of 158 468 matriculants (24%), 37 579 of 152 453 graduates (24%), 229 899 of 1 035 512 residents (22%), and 297 413 of 1 351 187 faculty members (26%). The mean (SD) RQ was significantly greater among Asian American residents (3.44 [0.15]) and faculty (3.54 [0.03]) compared with Asian applicants (3.3 [0.04]), matriculants (3.37 [0.03]), or graduates (3.31 [0.06]). Upon disaggregation, RQ was significantly lower among residents and faculty in 10 of 12 subgroups. Although subgroups, such as Taiwanese American, Indian American, and Chinese American, had RQs greater than 1 (eg, Chinese American graduates: mean [SD], RQ, 3.90 [0.21]), the RQs were less than 1 for Laotian, Cambodian, and Filipino American subgroups (eg, Filipino American graduates: mean [SD], RQ, 0.93 [0.06]) at almost every career stage. No significant RQ changes were observed over time for Laotian American and Cambodian American trainees, with a resident RQ of 0 in 8 of 25 and 4 of 25 specialties, respectively. Faculty RQ increased in 9 of 12 subgroups, but Cambodian American, Filipino American, Indonesian American, Laotian American, and Vietnamese American faculty (eg, Vietnamese American faculty: mean [SD], RQ, 0.59 [0.08]) had RQs less than 1.

**CONCLUSIONS AND RELEVANCE:**

In this cross-sectional study of Asian representation in US allopathic medical schools, Laotian American, Cambodian American, and Filipino American individuals were underrepresented at each stage of the physician workforce pathway. Efforts to promote diversity in medicine should account for these disparities to avoid perpetuating inequities.

## Key Points

**QUESTION:**

How does the representation of Asian American individuals in the US allopathic physician workforce vary when disaggregated by specific subgroups?

**FINDINGS:**

This cross-sectional study analyzed 94 934 medical school applicants, 39 849 matriculants, 37 579 graduates, 229 899 residents, and 297 413 faculty identifying as Asian between 2013 to 2021. While Asian American individuals overall were highly represented, Laotian, Cambodian, and Filipino American individuals were consistently underrepresented, especially in selective residency specialties.

**MEANING:**

These findings suggest that although Asian American individuals overall are not underrepresented in medicine, disaggregated analyses revealed substantial disparities, suggesting that diversity efforts should account for these differences to avoid perpetuating inequities in the physician workforce.

## Teaser

This cross-sectional study assesses representation by Asian race and disaggregated subgroups in the US allopathic medical school workforce using data from the Association of American Medical Colleges.

## Introduction

Anti-Asian sentiment during the COVID-19 pandemic underscored the ways in which systemic racism has harmed the health and advancement of Asian communities in the US.^1^ Asian American patients consistently report low-quality health encounters, with patient satisfaction scores up to 16% lower than those of non-Hispanic White patients.^2,3,4^ Meanwhile, monolithic conceptualizations of Asian race perpetuate aggregation in data collection and reporting, thereby masking the complex demographic, socioeconomic, genetic or ancestral, and cultural heterogeneity inherent to Asian populations. Limited available data suggest that some Asian subgroups prefer clinicians who demonstrate an understanding of their language, sociocultural practices, and/or religious beliefs.^5,6,7,8^ Moreover, for some subgroups, having a shared ethnicity or language with one’s physician is associated with improved communication and better adherence to care.^2,8,9^

Collectively, these data demonstrate how a more nationally representative physician workforce may help improve patient outcomes and promote equitable care. However, efforts to foster diversity in medicine have largely excluded Asian American individuals as they are not considered underrepresented in medicine (URM).^10,11^ In fact, Asian American individuals account for 1 in 5 US allopathic medical students and physicians,^12,13^ despite accounting for only 7% of the US population.^14^ Nevertheless, existing analyses of physician workforce representation have yet to disaggregate Asian race by subgroup—despite Asian American individuals tracing their ancestry to more than 40 countries of origin across the world’s most densely populated continent.

To establish a more accurate depiction of Asian American representation in medicine, we conducted a cross-sectional analysis of medical students, resident physicians, and academic faculty at US allopathic medical schools—with disaggregation by race, ethnicity, and familial country of origin. We hypothesized that while Asian American individuals are highly represented as a group, disparities in representation by country of origin become apparent upon disaggregation. Furthermore, although disparities in Asian American representation may be limited to certain subgroups during earlier stages of medical training, we hypothesized these disparities are more ubiquitous and pronounced across subgroups at later stages of career development.

## Methods

### Study Design, Data Source, and Participants

This study is a cross-sectional analysis of racial and ethnic representation among US allopathic medical school trainees (ie, applicants, matriculants, graduates, residents) and faculty between January 1, 2013, through December 31, 2021. Data were obtained via special reports generated by the Association of American Medical Colleges (AAMC) using multiple sources including the AAMC Applicant-Matriculant Data File, Student Records System, Graduate Medical Education Track Survey, and faculty roster. This cross-sectional study followed the Strengthening the Reporting of Observational Studies in Epidemiology (STROBE) reporting guideline. Because data were deidentified and did not include protected health information, formal institutional review board review and informed consent was not required per the Common Rule.

### Categorization of Self-Reported Race or Ethnicity

The primary exposure was self-reported race or ethnicity as per AAMC National Graduate Medical Education Census and US Census Bureau/Office of Management and Budget criteria. Individuals of Asian descent were further disaggregated by available self-reported familial country of origin (ie, Bangladeshi, Cambodian, Chinese, Filipino, Indian, Indonesian, Japanese, Korean, Pakistani, Taiwanese, and Vietnamese). Individuals who reported other or unknown race or Asian race with no or more than 1 subgroup classification were excluded from analyses (eFigure 1 in Supplement 1).

### Categorization of Non–US Citizens or Nonlegal Permanent Residents

Data for noncitizen or non-legal permanent resident (non-LPR) status were available for US allopathic medical school applicants, matriculants, graduates and resident physicians, but not faculty. Per AAMC policy, details on self-reported race or ethnicity of noncitizens or non–LPRs were unavailable.

### Representation Quotient

The primary outcome measure was the representation quotient (RQ): defined as the proportion of individuals who identify as a specific racial or ethnic group within a population of interest (ie, applicants, matriculants, graduates; residents; faculty), divided by the estimated proportion of that racial or ethnic group within the US population of a similar age in a given year according to US census data.^12^ RQ values equal to 1 denote representation in the population of interest that is equivalent to that group’s representation in the total US population. RQ values greater than or less than 1 denote representation that is higher or lower than expected, respectively, based on a given group’s representation in the total US population. An RQ of 0 denotes no representation.

### Statistical Analyses

We first analyzed our data descriptively and calculated the RQ at a population-level for Asian American individuals in aggregate and by subgroup for each career stage. One-way analysis of variance (ANOVA) was used to assess differences in mean RQ by career stage for Asian American individuals in aggregate and by subgroup. To examine whether the magnitude of representation differed at later career stages, we performed post-hoc analyses using the Tukey honestly significant differences (HSD) test, which accounts for multiple pairwise comparisons.

We used separate linear regression models to estimate trends in RQ over time at each career stage for Asian American individuals in aggregate and by subgroup, reporting RQ slope estimates along with 95% CIs. We set statistical significance at *P* = .05 for our aggregate Asian American trends analysis and *P* = .004 for the disaggregated subgroup analysis, using Bonferroni correction to account for multiple testing for each of the 12 subgroups. All analyses were conducted using R version 4.0.3 (R Project for Statistical Computing). Data were analyzed between March and May 2024, and all tests were 2-sided.

## Results

A total of 385 775 applicants, 158 468 matriculants, 152 453 graduates, 1 035 512 residents, and 1 351 187 faculty enrolled or employed at US allopathic medical schools between 2013 and 2021 met criteria for study inclusion. Asian American individuals accounted for 94 934 of 385 775 applicants (23%), 39 849 of 158 468 matriculants (24%), 37 579 of 152 453 graduates (24%), 229 899 of 1 035 512 residents (22%), and 297 413 of 1 351 187 faculty members (26%) (Table 1). Although individuals of Indian descent were the largest Asian subgroup by count and accounted for 28 142 of all Asian applicants (31%), 12 555 matriculants (33%), 12 136 graduates (32%), 88 162 residents (37%), and 57 217 faculty members (27%), Taiwanese individuals were the most highly represented by mean (SD) RQ (applicants, 17.0 [2.51]; matriculants, 19.4 [3.02]; graduates, 21.8 [1.86]; residents, 13.9 [3.13]; faculty, 1.30 [1.19]) (Table 1). Conversely, individuals of Laotian descent were the smallest subgroup by both count and RQ, accounting for less than 1% of Asian American individuals at all levels (Table 1).

**Table 1.** Counts, Proportions, and Representation Quotient by Career Stage and Asian Ethnic Subgroup

Characteristic	Career stage									
	Applicants		Matriculants		Graduates		Residents		Faculty	
	No. (%)	RQ, mean (SD)	No. (%)	RQ, mean (SD)	No. (%)	RQ, mean (SD)	No. (%)	RQ, mean (SD)	No. (%)	RQ, mean (SD)
Total	385 775	NA	158 468	NA	152 453	NA	1 035 512	NA	1 351 187	NA
Asian	94 934 (24.6)	3.30 (0.04)	39 849 (25.1)	3.37 (0.03)	37 579 (24.7)	3.31 (0.06)	229 899 (22.2)	3.44 (0.15)	297 413 (22.0)	3.54 (0.03)
Noncitizen/non–LPR	15 429 (4.0)	0.36 (0.01)	2265 (1.4)	0.13 (0.01)	2488 (1.6)	0.15 (0.02)	153 079 (14.8)	1.18 (0.09)	NA	NA
Asian subgroup^a^										
Total	94 934	NA	38 849	NA	37 579	NA	229 899	NA	297 413	NA
Bangladeshi	1831 (1.9)	7.07 (1.19)	671 (1.7)	6.15 (1.06)	560 (1.3)	5.38 (0.87)	3163 (1.4)	4.74 (1.13)	1269 (0.4)	1.85 (0.08)
Cambodian	512 (0.5)	1.07 (0.10)	141 (0.4)	0.70 (0.15)	107 (0.3)	0.55 (0.17)	511 (0.2)	0.42 (0.12)	738 (0.2)	0.66 (0.21)
Chinese	19 735 (20.8)	3.20 (0.34)	9833 (24.7)	3.77 (0.34)	9805 (26.1)	3.90 (0.21)	47 691 (20.7)	3.26 (0.17)	46 472 (15.6)	2.65 (0.07)
Filipino	6441 (6.8)	1.32 (0.14)	2037 (5.1)	0.99 (0.14)	1830 (4.9)	0.93 (0.06)	11 376 (4.9)	0.93 (0.02)	8472 (2.8)	0.64 (0.05)
Indian	28 142 (29.6)	4.53 (0.23)	12 555 (31.5)	4.78 (0.22)	12 136 (32.3)	4.87 (0.46)	88 162 (38.3)	5.26 (0.73)	NA	NA
Indian/Pakistani	NA	NA	NA	NA	NA	NA	NA	NA	84 880 (28.5)	3.06 (0.19)
Indonesian	481 (0.5)	2.81 (0.43)	195 (0.5)	2.71 (0.67)	187 (0.5)	2.68 (0.76)	734 (0.3)	1.68 (0.67)	103 (<0.1)	0.30 (0.25)
Japanese	3621 (3.8)	2.56 (0.20)	1150 (3.8)	2.53 (0.18)	1475 (3.9)	2.57 (0.28)	7071 (3.1)	1.89 (0.17)	5729 (1.9)	1.41 (0.17)
Korean	10 158 (10.7)	4.07 (0.12)	4156 (10.4)	3.94 (0.17)	3883 (10.3)	3.83 (0.26)	18 751 (8.2)	2.99 (0.11)	14 754 (5.0)	2.19 (0.25)
Laotian	184 (0.2)	0.49 (0.10)	53 (0.1)	0.33 (0.15)	44 (0.1)	0.29 (0.15)	164 (0.1)	0.17 (0.08)	9 (<0.1)	0.01 (0.01)
Pakistani	6377 (6.7)	9.18 (1.05)	1958 (4.9)	7.01 (1.13)	1856 (4.9)	6.60 (0.70)	18 313 (8.0)	11.0 (0.37)	NA	NA
Taiwanese	4942 (5.2)	17.0 (2.51)	1672 (4.2)	19.4 (3.02)	2587 (6.9)	21.8 (1.86)	10 828 (4.7)	13.9 (3.13)	1093 (0.4)	1.30 (1.19)
Vietnamese	8478 (8.9)	3.30 (0.22)	2064 (5.2)	2.76 (0.20)	2841 (7.6)	2.72 (0.24)	14 011 (6.1)	2.24 (0.27)	5052 (2.4)	0.59 (0.08)

Abbreviations: LPR, legal permanent resident; NA, not applicable; RQ, representation quotient.

^a^ Counts for each subgroup are alone or in combination with another subgroup and/or race or ethnicity.

### Representation by Career Stage and Asian Subgroup

**Figure 1.**
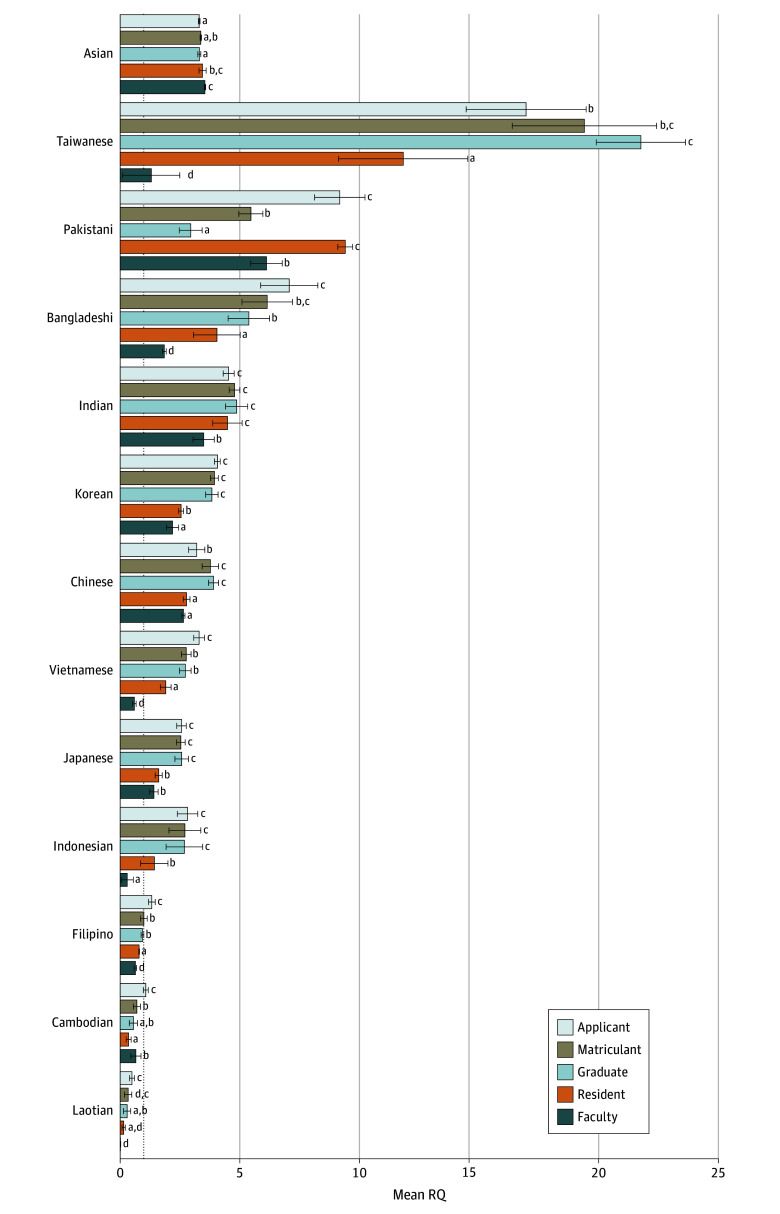
Representation by Asian Ethnic Subgroup and Career Stage, 2013-2021 This stacked bar histogram displays the mean representation quotients (RQs) for Asian subgroups at different stages of the medical career pathway: applicant, matriculant, graduate, resident, and faculty. RQs indicate the representation of each subgroup relative to their proportion in the US population, where RQ of 1 signifies equivalent representation, RQ greater than 1 indicates higher representation, and RQ less than 1 indicates lower representation. Superscript letters (a, b, c, d, e) designate statistical significance per 1-way analysis of variance and Tukey honestly significance difference test. Subgroups sharing the same letter are not significantly different from each other, while those with different letters are significantly different (*P* < .05). For example, among Taiwanese individuals, there was no statistically significant difference between the RQs of matriculants and applicants and graduates, but there was a statistically significant difference between applicants and graduates.

The directionality of the association between RQ and career stage varied among Asian American individuals when assessed in aggregate vs by subgroup (Figure 1). On post hoc analysis, although the mean (SD) RQ did not significantly differ between Asian applicants (3.30 [0.04]), matriculants (3.37 [0.03]), or graduates (3.31 [0.06]) in aggregate, the mean RQ among Asian American residents (3.44 [0.15]) was significantly greater than that of applicants (Tukey HSD, *P* = .005) and graduates (*P* = .009). Likewise, mean RQ among Asian American faculty was significantly greater than that of applicants, matriculants, and graduates (all *P* < .001). There was no significant difference in mean RQ were observed between Asian American residents and faculty.

In contrast, upon disaggregation, representation was generally lower (as opposed to higher) at later career stages (Figure 1 and Table 1). For instance, pairwise comparisons of Taiwanese American applicants vs matriculants (mean RQ [SD], 17 [2.51] vs 19.4 [3.02]; Tukey HSD, *P* = .25) and matriculants vs graduates (7 [2.5] vs 21.8 [1.86]; *P* = .28) did not reveal significant differences; however, mean (SD) RQ was significantly lower among Taiwanese American residents (13.9 [3.13]) and faculty (1.3 [1.19]) (*P* < .001 for all resident or faculty pairwise comparisons). This pattern was seen in 10 of 12 (83%) Asian American subgroups, of which, 5 (50%) had a mean (SD) faculty RQ less than 1 (Cambodian American faculty, 0.66 [0.21]; Filipino American faculty, 0.64 [0.05]; Indonesian American faculty, 0.3 [0.25]; Laotian American faculty, 0.01 [0.01]; Vietnamese American faculty, 0.59 [0.08]). Notably, while mean RQ was greater than 1 for Indonesian American and Vietnamese American individuals in medical school and residency, mean RQ was less than 1 for Laotian American, Cambodian American, and Filipino American individuals at nearly every stage (Figure 1 and Table 1).

### Resident Representation by Specialty

**Figure 2.**
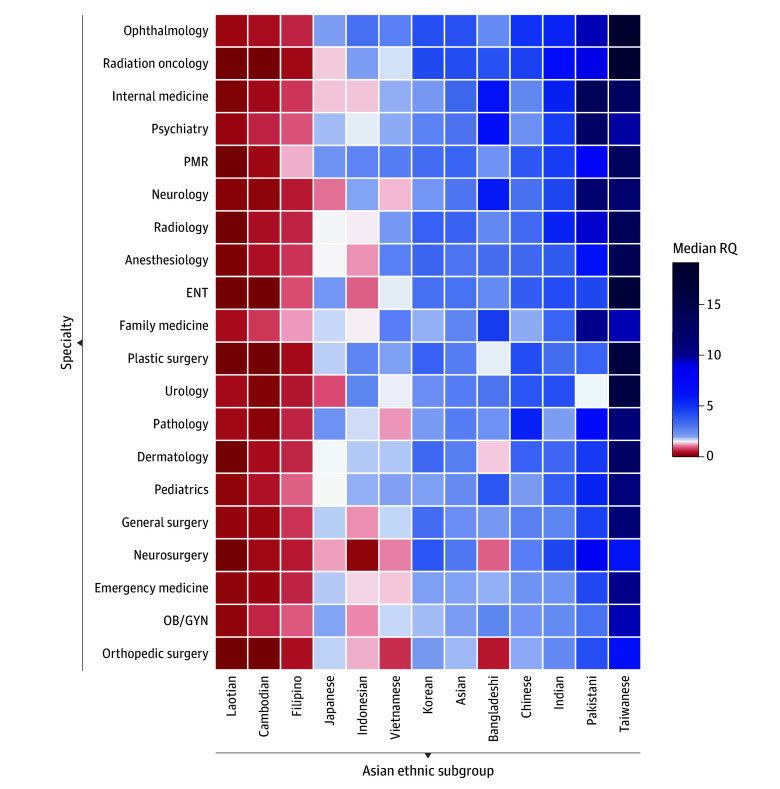
Representation by Asian Ethnic Subgroup and Residency Specialty, 2013-2021 This heatmap displays the median RQs for various Asian ethnic subgroups across different medical specialties. RQs represent the ratio of each subgroup’s representation in a given specialty compared to their proportion in the US population, where RQ of 1 indicates equivalent representation, RQ greater than 1 indicates higher representation, and RQ less than 1 indicates lower representation. ENT indicates ear, nose, and throat; OB/GYN, obstetrics and gynecology; PMR, physical medicine and rehabilitation; RQ, representation quotient.

An RQ heatmap demonstrating representation among Asian resident physicians by specialty and Asian subgroup is presented in Figure 2. In aggregate, Asian American individuals (either alone or in combination with another race or ethnicity) were highly represented across specialties. The 3 specialties with the highest median (IQR) RQ values were radiation oncology (4.19 [3.97-4.43]), ophthalmology (4.08 [4.01-4.17]), and internal medicine (3.48 [3.28-3.62]). The 3 specialties with the lowest were orthopedic surgery (1.86 [1.71-1.94]), obstetrics and gynecology (2.09 [2.06-2.12]), and emergency medicine (2.00 [1.97-2.01]).

On subgroup analysis, Taiwanese American, Pakistani American, and Indian American residents had the highest median (IQR) RQ values across specialties (Figure 2). Conversely, Laotian American, Cambodian American, and Filipino American residents had RQ values less than 1, with the exception of Filipino residents in family medicine (1.20 [1.17-1.38]) and pediatrics (1.28 [1.17-1.38]) (Figure 2 and eTable 1 in Supplement 1). Notably, Laotian American and Cambodian American residents had an RQ of 0 in 8 of 25 and 4 of 25 specialties (Figure 2 and eTable 1 in Supplement 1).

### Trends in Representation by Career Stage and Asian Subgroup

#### Applicants, Matriculants, and Graduates

Trends in representation of Asian American individuals by career stage in aggregate and by subgroup are presented in Table 2 and eFigure 2 in Supplement 1. From 2013 to 2021, representation of Asian American individuals increased by an absolute 5 percentage points among applicants (20% to 25%), 4 percentage points among matriculants (21% to 25%), and 3 percentage points among graduates (23% to 26%); however, there was no corresponding change in mean applicant, matriculant, or graduate RQ (Table 2). Upon disaggregation, mean RQ significantly increased among Filipino American applicants (RQ slope, 0.05 [95% CI, 0.03-0.08]; adjusted *P* = .036), Filipino American matriculants (0.05 [95% CI, 0.03-0.07]; adjusted *P* = .012), and Vietnamese American graduates (0.09 [95% CI, 0.06-0.12]; adjusted *P* = .012). No other significant changes in mean applicant, matriculant, or graduate RQ were observed (Table 2).

**Table 2.** RQ Trends by Career Stage and Asian Ethnic Subgroup, 2013 to 2021^a^

Group	Applicants (n = 94 934)		Matriculants (n = 38 849)		Graduates (n = 37 579)		Residents (n = 229 899)		Faculty (n = 297 413)	
	RQ slope (95% CI)	*P* value	RQ slope (95% CI)	*P* value	RQ slope (95% CI)	*P* value	RQ slope (95% CI)	*P* value	RQ slope (95% CI)	*P* value
Asian	0.01 (0.004 to 0.02)	.01	0.007 (0.004 to 0.01)	<.001	0.01 (0.008 to 0.02)	<.001	−0.06 (−0.07 to −0.05)	<.001	−0.006 (−0.02 to 0.003)	.15
Noncitizen or non–LPR	−0.0002 (−0.005 to 0.004)	.91	−0.001 (−0.006 to 0.003)	.52	−0.006 (−0.012 to 0.000006)	.05	0.02 (−0.01 to 0.05)	.21	NA	NA
Asian subgroup^b^										
Bangladeshi	0.42 (0.14 to 0.59)	.005	0.36 (0.14 to 0.59)	.007	0.22 (−0.04 to 0.48)	.08	0.35 (0.16-0.54)	.004	0.01 (−0.02 to 0.04)	.38
Cambodian	0.02 (−0.02 to 0.06)	.31	0.02 (−0.04 to 0.08)	.40	0.04 (−0.007 to 0.10)	.08	0.04 (0.03-0.05)	<.001	0.08 (0.06 to 0.11)	<.001
Chinese	−0.10 (−0.15 to −0.04)	.005	−0.10 (−0.17 to −0.03)	.01	−0.07 (−0.11 to −0.03)	.005	−0.05 (−0.08 to −0.03)	.002	0.02 (0.0006 to 0.04)	.05
Filipino	0.05 (0.03 to 0.08)	.003	0.05 (0.03 to 0.07)	<.001	0.01 (−0.004 to 0.03)	.11	0.003 (−0.002 to 0.008)	.17	0.02 (0.01 to 0.03)	<.001
Indian	0.03 (−0.07 to 0.13)	.49	0.06 (−0.03 to 0.16)	.17	−0.10 (−0.248 to 0.06)	.18	−0.24 (−0.32 to −0.15)	<.001	0.17 (0.11 to 0.23)	<.001
Indonesian	0.13 (0.02 to 0.23)	.03	0.18 (0.005 to 0.36)	.05	0.21 (0.004 to 0.42)	.05	0.23 (0.19 to 0.27)	<.001	0.10 (0.07 to 0.13)	<.001
Japanese	0.05 (−0.01 to 0.10)	.09	0.05 (0.01 to 0.10)	.02	0.09 (0.03 to 0.15)	.01	0.05 (0.01 to 0.08)	.01	0.07 (0.05 to 0.08)	<.001
Korean	0.04 (−0.004 to 0.08)	.07	0.06 (0.01 to 0.11)	.03	0.08 (0.01 to 0.14)	.03	0.03 (−0.001 to 0.06)	.06	0.1 (0.09 to 0.12)	<.001
Laotian	0.03 (0.002 to 0.05)	.04	0.01 (−0.04 to 0.07)	.60	0.04 (−0.005 to 0.09)	.07	0.03 (0.02 to 0.03)	<.001	0.004 (0.002 to 0.007)	.008
Pakistani	0.28 (−0.08 to 0.63)	.10	0.34 (0.02 to 0.66)	.04	0.19 (0.004 to 0.38)	.05	−0.02 (−0.15 to 0.10)	.65	0.25 (0.16 to 0.35)	<.001
Taiwanese	−0.67 (−1.21 to −0.12)	.02	−0.82 (−1.55 to −0.09)	.03	−0.62 (−1.01 to −0.23)	.008	0.85 (0.15 to 1.55)	.02	0.47 (0.34 to 0.6)	<.001
Vietnamese	0.07 (0.007 to 0.14)	.03	0.05 (−0.02 to 0.12)	.13	0.089 (0.06 to 0.12)	<.001	0.08 (0.07 to 0.11)	<.001	0.02 (0.01 to 0.03)	<.001

Abbreviations: LPR, legal permanent resident; NA, not applicable; RQ, representation quotient.

^a^ Threshold for significance set at *P* = .004 using Bonferroni correction for multiple comparisons.

^b^ Counts for each subgroup are alone or in combination with another subgroup and/or race or ethnicity.

#### Residents and Faculty

From 2013 to 2021, the absolute proportion of resident physicians of who reported Asian ancestry (alone or in combination) remained unchanged at 22%; however, representation of Asian American individuals significantly decreased by median RQ from 2013 to 2021 (3.66 to 3.26; RQ slope, −0.06 [95% CI, −0.07 to −0.04]; *P* < .001) (Table 2). On subgroup analysis, representation significantly increased among Cambodian American residents in 4 specialties; Filipino American residents in 2 specialties; and Laotian American residents in 2 specialties (eTable 2 in Supplement 1). Across 25 specialties, significant increases in representation were also observed among resident physicians of Bangladeshi (2 specialties [8%]), Vietnamese (4 specialties [16%]), Indonesian (5 specialties [20%]), and Taiwanese (6 specialties [24%]) descent; while concomitant declines in representation were observed among resident physicians of Indian (10 specialties [40%]), Chinese (3 specialties [12%]), Korean (2 specialties [8%]), and Pakistani (1 specialty [4%]) descent (eTable 2 in Supplement 1).

Although the proportion of faculty reporting Asian ancestry increased by 4 percentage points from 2013 to 2021 (20% to 24%), no significant change in mean RQ was observed (RQ slope, 3.59 to 3.55; −0.006 [95% CI, −0.015 to 0.003]; *P* = .15). All Asian American subgroups had significant increases in faculty RQ, except for faculty of Laotian descent (0.004 [95% CI, 0.002 to 0.007]; *P* = .008) (Table 2).

### Representation of Non–US Citizens or Nonlegal Permanent Residents

Non–US citizens and non–LPRs accounted for 15 429 (4%) of applicants, 2265 (1.4%) of matriculants, 2488 (1.6%) of graduates, and 153 079 (14.8%) of resident physicians at US allopathic medical schools (Table 1). Mean RQ was less than 1 among applicants (mean [SD] RQ, 0.36 [0.01]), matriculants (0.13 [0.01]), and graduates (0.15 [0.01]), but significantly higher among residents (1.18 [0.09]). There were no significant trends in non–US citizen or non–LPR representation among applicants, matriculants, graduates, or residents during the study period (Table 2).

## Discussion

To our knowledge, this nationally comprehensive study of representation among Asian American individuals in medicine is the first and largest study of its kind to date. Our study suggests that while Asian American individuals were highly represented overall, the degree of representation varied by subgroup. Whereas individuals of Taiwanese, Pakistani, Korean, and Chinese descent had consistently high levels of representation, Southeast Asian American individuals—namely, those of Laotian, Cambodian, and Filipino descent—were underrepresented at nearly every stage of the physician workforce pathway, with Laotian American individuals faring the worst. Although Cambodian American and Filipino American representation increased during the study period, parallel improvements in Laotian American representation were not observed. Additionally, Laotian American, Cambodian American, and Filipino American residents were underrepresented across multiple specialties, especially in selective specialties. Finally, for nearly all Asian American subgroups, representation diminished with each successive career stage; however, this only became apparent upon disaggregation.

Several factors at multiple levels—societal, institutional, and individual—likely contribute to these findings, which should be interpreted in the larger sociohistorical context and not exclusively through a cultural lens that relies on racist stereotypes of superior motivation and effort. The overrepresentation of certain Asian American subgroups likely reflects major changes to immigration policy, which changed the occupational and educational backgrounds of Asian immigrants. The 1965 Immigration and Nationality Act lifted country-based immigration quotas and prioritized skill-based visas, which facilitated the hyperselective migration of individuals from disproportionately science, technology, engineering, and mathematics (STEM) backgrounds with college graduation rates exceeding both the populations of the origin country and the US.^15^ In the US, immigrants formed ethnic enclaves and social networks that produced community-based resources to supplement formal education as a response to residential segregation and racial discrimination in the labor market. The result was that second-generation children were able to attain upward socioeconomic mobility because they benefited from ethnic social capital in the form of after-school programs, SAT preparation, and tutoring before college.^16,17^ At the family and individual level, there was also some evidence that the parental decision to steer their children toward medicine and other STEM fields reflects a belief that the successful attainment of hard skills may shield them from racial discrimination in the workplace.^16^

In contrast, the same period saw the forced migration and resettlement of Vietnamese, Laotian, Hmong, and Cambodian peoples as a direct result of US policy in the Vietnam War and ushered in a marked increase in ethnic diversity and socioeconomic stratification in the Asian American population. Today, income inequality among Asian American individuals exceeds that of other major racial and ethnic groups in the US, with a disproportionate number of Laotian American and Cambodian American individuals living below the poverty line.^18,19^ As a result, Laotian American and Cambodian American youths were more likely to attend underresourced kindergarten to 12th grade schools, face the competing demands of helping support their families financially, have lower high school graduation rates, and encounter negative stereotyping from teachers, all of which may deter them from attending college and ultimately applying to medical school.^20^ Cambodian American and Laotian American individuals likely face structural barriers at the medical school application stage similar to other underrepresented groups in medicine due to the upfront time and financial investment, such as outstanding and large levels of college debt, the inability to afford test preparation materials, and limited opportunities for exposure to science and medicine.^21^ Therefore, our findings of underrepresentation for these subgroups were unsurprising given the lack of socioeconomic diversity in US medical schools and mirror a broader pattern of exclusion of Southeast Asian students in STEM.^22^

Our findings for Filipino American individuals may appear surprising given the higher median household income and parental educational attainment of this group. However, these results were consistent with prior data showing that second-generation Filipino American individuals were less likely to pursue graduate education compared with their parents, other Asian American individuals, and White students.^15,23^ Although the underlying reasons were likely multifactorial, this pattern of downward intergenerational mobility may exist, in part, as a consequence of American colonialism. The US remade the Philippine education system with English as the primary language during the colonial period and developed nursing schools to establish a steady flow of Filipino nurses to address US workforce shortages.^24,25^ Such policy has had a lasting impact on the Philippine diaspora, as the Philippines is the leading exporter of nurses globally, an occupational preference transmitted to US-born children. Qualitative data suggested that Filipino American parents encourage their children toward careers in nursing and other allied health professions, internalizing a colonial mentality that Filipino individuals are well-suited for these occupations. Additionally, Filipino American students were often racialized as Latino in high school, perceived as less smart than East Asian individuals, and thus less likely to benefit from the model minority myth and be funneled into higher level science and math classes. Moreover, Filipino American parents typically viewed pursuing a career in medicine as detracting from familial obligations and financially risky due to the high debt and delayed return on investment compared with nursing.^26^

Underrepresentation of Laotian American, Cambodian American, and Filipino American individuals across residency specialties, especially the most selective ones with the longest training, indicated that increasing representation of medical school student bodies alone may be insufficient. Like first-generation, low-income (FGLI) students, Southeast Asian American students were more likely to have parents without advanced degrees, which could lead to several disadvantages, including limited guidance navigating academic challenges, fewer professional connections and mentors, and less familiarity with potential career pathways.^22,27,28^ Societal expectations of the model minority myth further complicates their experiences via stereotype threat and implicit bias.^10^ Overreliance on resilience to overcome unsupportive learning environments may discourage the seeking of help or mentorship.^29,30^ The lack of racial and ethnic diversity in selective specialties,^31^ bias in core clerkship evaluations,^32^ and exclusion from accolades, such as Alpha Omega Alpha,^33^ limit opportunities to pursue careers in more selective specialties. Therefore, medical schools can inadvertently function as racialized organizations and reproduce the underrepresentation of Southeast Asian American students through occupational segregation.^34^

Proactive measures are likely needed to promote inclusion and facilitate success of excluded Asian subgroups at every career stage. Examples include targeted interventions to address income inequality, educational support for students from refugee communities, and enrichment programs offering mentorship and shadowing opportunities for FGLI students.^28,35^ Additionally, while medical schools should continue efforts to employ holistic review to account for distance traveled,^36,37^ they should also consider broadening the URM definition to include underrepresented Asian American subgroups.^38^ This may be especially impactful for more populous and diverse states where medical school composition is paradoxically less representative.^12^

Importantly, our findings that some subgroups are overrepresented among students and residents should not imply that these groups should face a higher bar in the admissions process. Instead, these findings underscore the possible career outcomes for underrepresented groups with targeted investment in institutions and programs to address structural barriers at each stage of the physician training pathway.^35^ Moreover, our finding of diminishing representation, including at the faculty level, is in line with prior evidence that Asian American individuals experience a glass ceiling that excludes them from medical leadership and senior faculty, in a way similar to other industries.^11,39,40^ Inclusion in structured mentorship and coaching initiatives for junior faculty could help foster and sustain a more diverse leadership pool.^41,42^

Taken together, our findings added to the nascent but growing body of literature challenging monolithic conceptualizations of Asian American individuals in the US and in medicine. Disaggregated data, as presented in this article, can both unmask disparities and facilitate efforts to promote equity and diversity in medicine. A continued lack of disaggregation may not only entrench existing disparities,^43,44^ but may also further compound underinvestment in Asian American communities: only 0.17% of National Institutes of Health funding has supported studies on Asian American, Native Hawaiian, or Pacific Islander individuals’ health over the last 25 years.^45^

In the wake of the 2023 Supreme Court ruling banning affirmative action, students may be less inclined or able to disclose their race or ethnicity. Thus, efforts to collect and report data on representation in medicine may prove more challenging moving forward.^46^ Given that the AAMC began reliably disaggregating data by Asian American subgroup in 2013, our study may represent the only contemporary snapshot of Asian American representation in medicine for the foreseeable future. Nevertheless, our findings warrant further research, including intersectional, longitudinal, and qualitative studies to identify factors associated with (and reasons underlying) attrition vs retention of excluded Asian American subgroups.

### Limitations

This study has several limitations. First, AAMC data collection methods and subsequent exclusion of individuals with multiple, missing, or unreported racial or ethnic identities may bias results toward underrepresentation. However, sensitivity analyses, including Asian race in combination with other racial or ethnic categories, did not meaningfully alter our findings. Second, AAMC special reports do not include certain variables, including information on other marginalized Asian ethnic groups (eg, Hmong). Likewise, the AAMC faculty roster does not collect information on citizenship/immigration status; however, the potential impact is likely minimal since faculty are more likely to be citizens or LPRs. Finally, these data do not distinguish between domestic and international medical graduates, and they only include data for individuals with medical doctor (ie, MD) degrees and exclude other medical professionals, such as doctors of osteopathic medicine (ie, DO), thus limiting generalizability.

## Conclusions

In this cross-sectional study of Asian American representation in US allopathic medical schools, Southeast Asian individuals, particularly those of Laotian, Cambodian, and Filipino descent, were underrepresented at nearly every stage of the physician workforce pathway. Our findings added to a growing body of literature challenging monolithic conceptualizations of Asian race in the US and in medicine. Although additional research is warranted, efforts to promote diversity in medicine should account for these disparities to avoid perpetuating inequities and build a more inclusive health care workforce.

## Notes

Supplement 1. **eFigure 1.** CONSORT Diagram **eFigure 2. **Asian American Representation by Career Stage and Asian Subgroup, 2013-2021 **eTable 1.** Resident Representation by Specialty and Asian Subgroup, 2013-2021 **eTable 2.** Resident RQ Trends by Specialty and Asian Subgroup, 2013 to 2021

Supplement 2.
Data Sharing Statement
