# Supplementary material for: Asian American Representation in Medicine by Career Stage and Residency Specialty
Source: JAMA Netw Open. 2024 Nov 19;7(11):e2444478. doi: 10.1001/jamanetworkopen.2024.44478 (PMC11577148; doi:10.1001/jamanetworkopen.2024.44478)
Supplement: Supplement 1. — eFigure 1. CONSORT Diagram eFigure 2. Asian American Representation by Career Stage and Asian Subgroup, 2013-2021 eTable 1. Resident Representation by Specialty and Asian Subgroup, 2013-2021 eTable 2. Resident RQ Trends by Specialty and Asian Subgroup, 2013 to 2021 [file jamanetwopen-e2444478-s001.pdf]

Supplemental Online Content

Santos PMG, Oronce CIA, Shah K, etc. Asian American representation in medicine by career stage and residency specialty. *JAMA Netw Open*. 2024;7(11):e2444478. doi:10.1001/jamanetworkopen.2024.44478

eFigure 1. CONSORT Diagram
eFigure 2. Asian American Representation by Career Stage and Asian Subgroup, 2013-2021
eTable 1. Resident Representation by Specialty and Asian Subgroup, 2013-2021
eTable 2. Resident RQ Trends by Specialty and Asian Subgroup, 2013 to 2021

This supplemental material has been provided by the authors to give readers additional information about their work.

© 2024 Santos PMG et al. *JAMA Network Open.*

eFigure 1. **CONSORT Diagram**

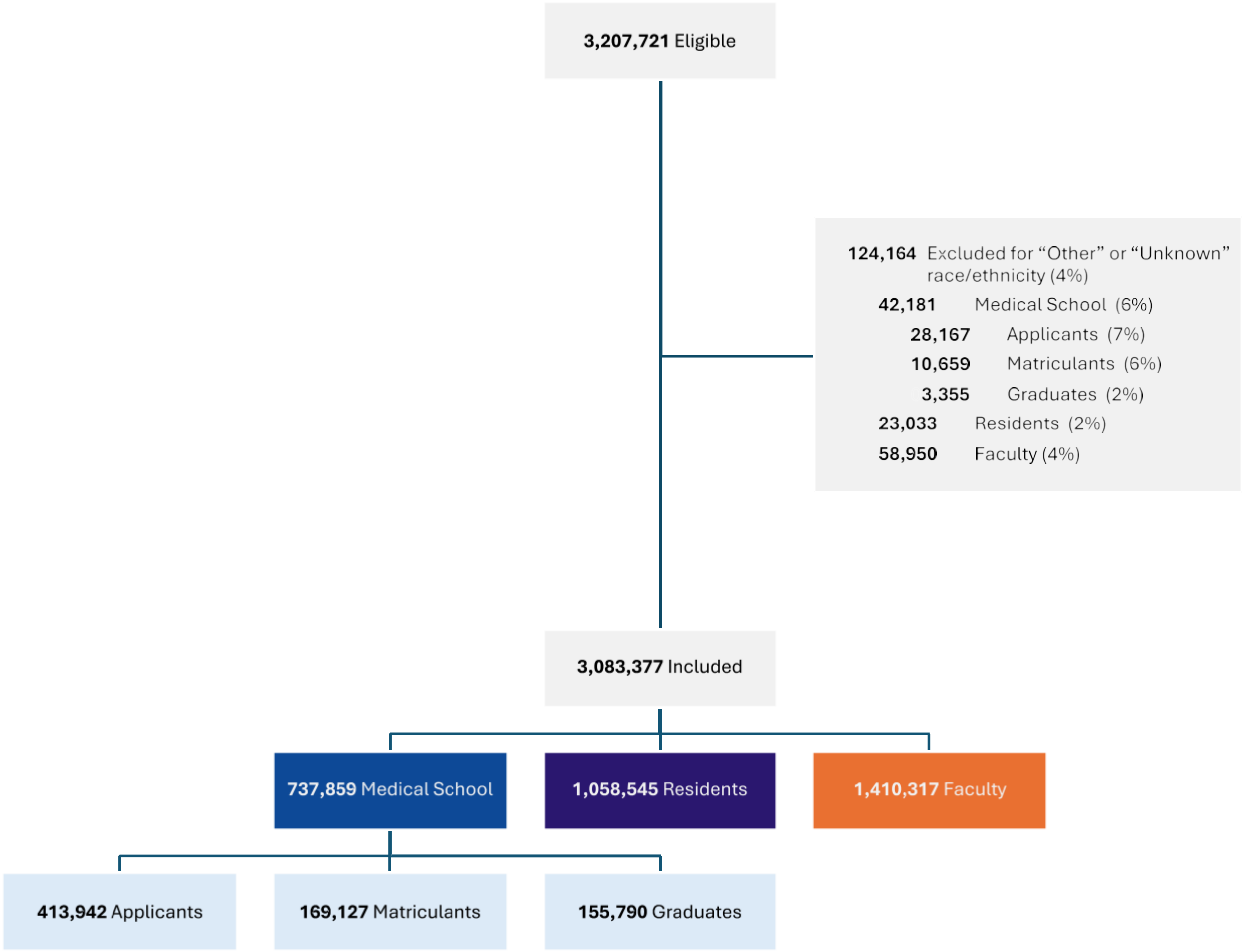

© 2024 Santos PMG et al. *JAMA Network Open.*

eFigure 2. **Asian American Representation by Career Stage and Asian Subgroup, 2013-2021**

A

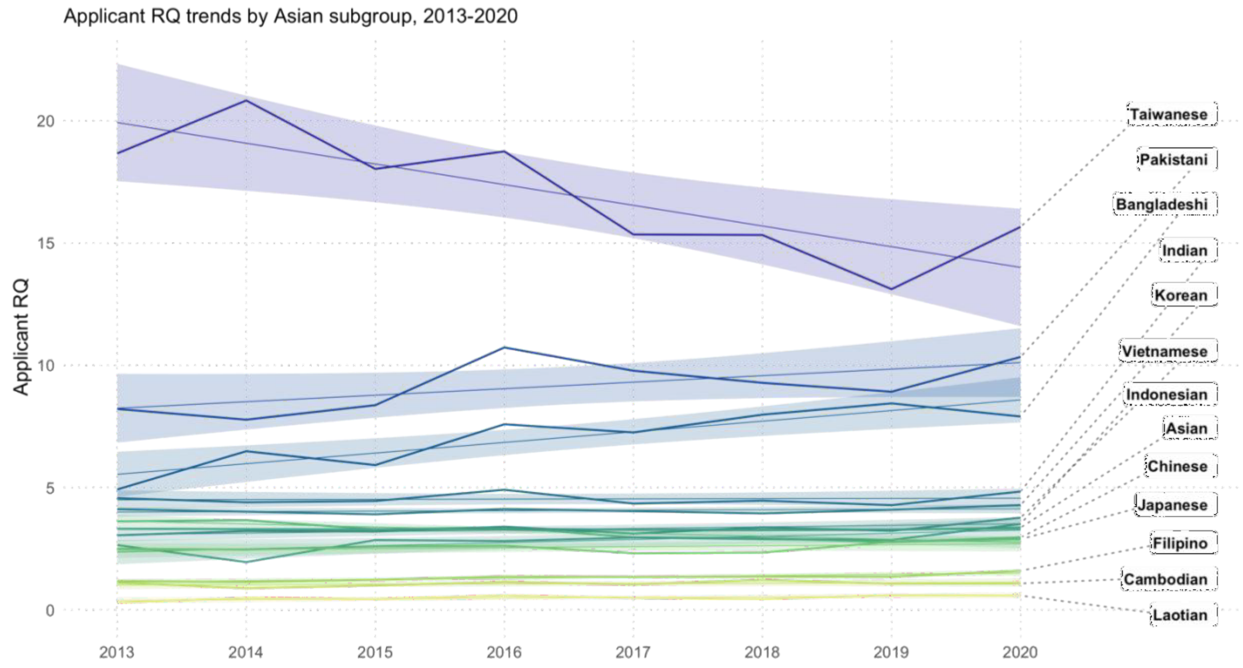

B

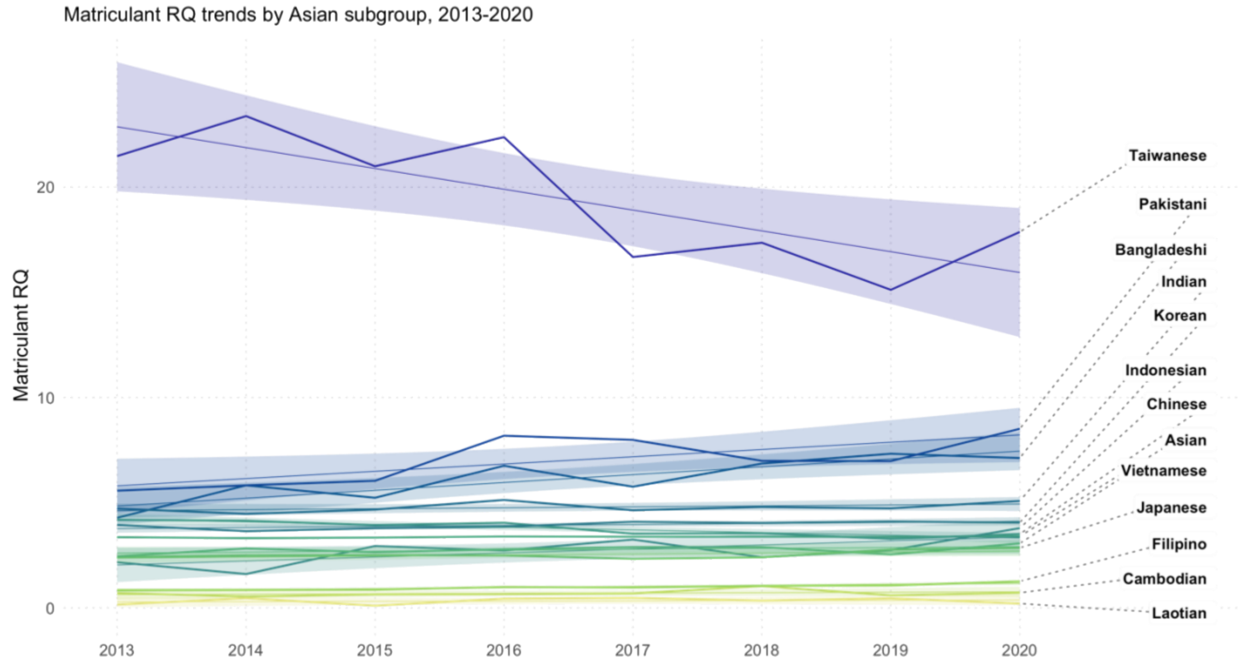

Abbreviation: RQ, Representation Quotient

© 2024 Santos PMG et al. *JAMA Network Open.*

eFigure 2. Asian American Representation by Career Stage and Asian Subgroup, 2013-2021 (continued)

C

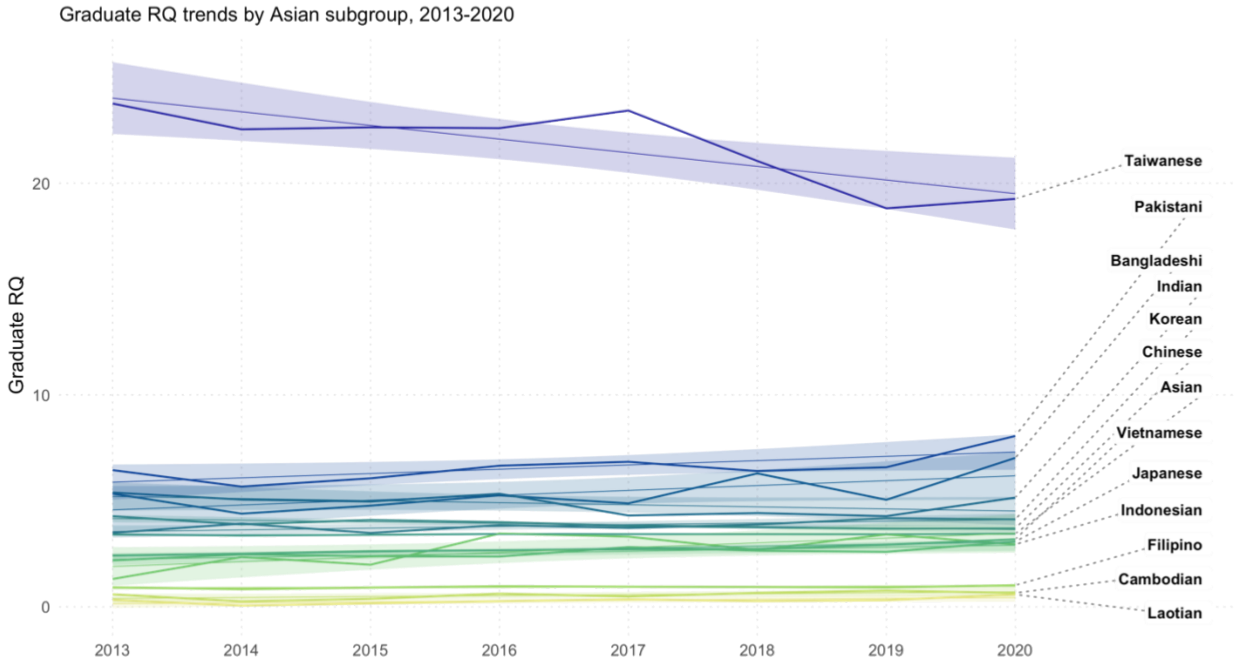

D

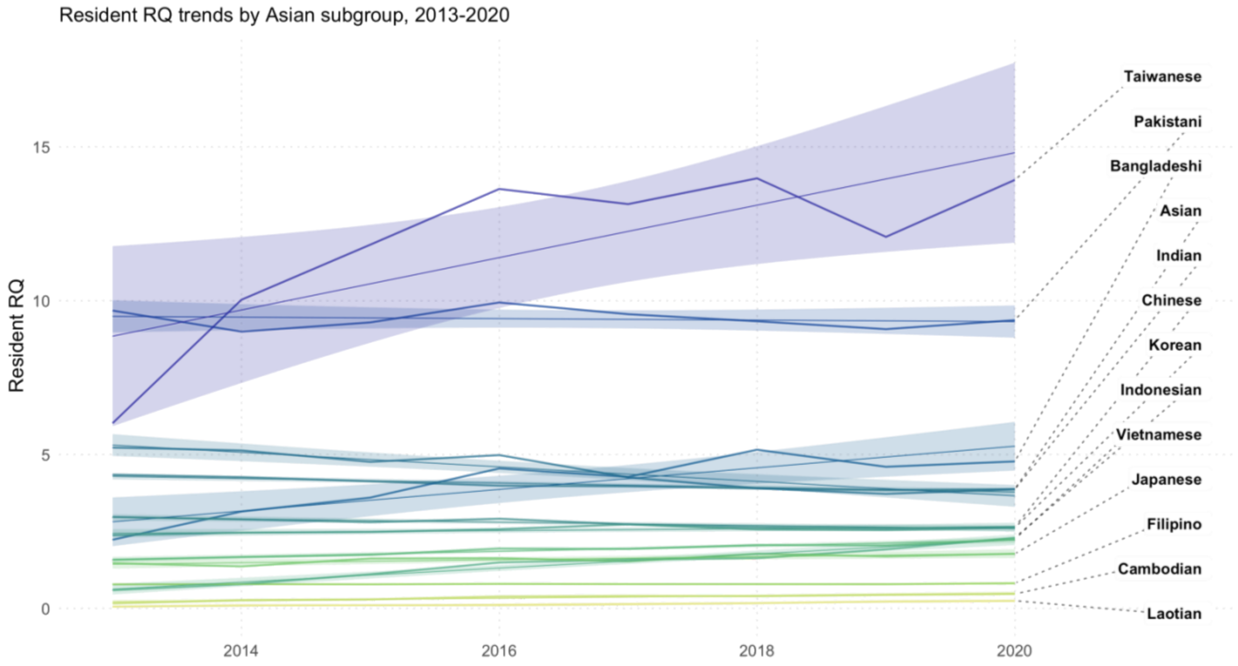

Abbreviation: RQ, Representation Quotient

© 2024 Santos PMG et al. *JAMA Network Open.*

eFigure 2. Asian American Representation by Career Stage and Asian Subgroup, 2013-2021 (continued)

E

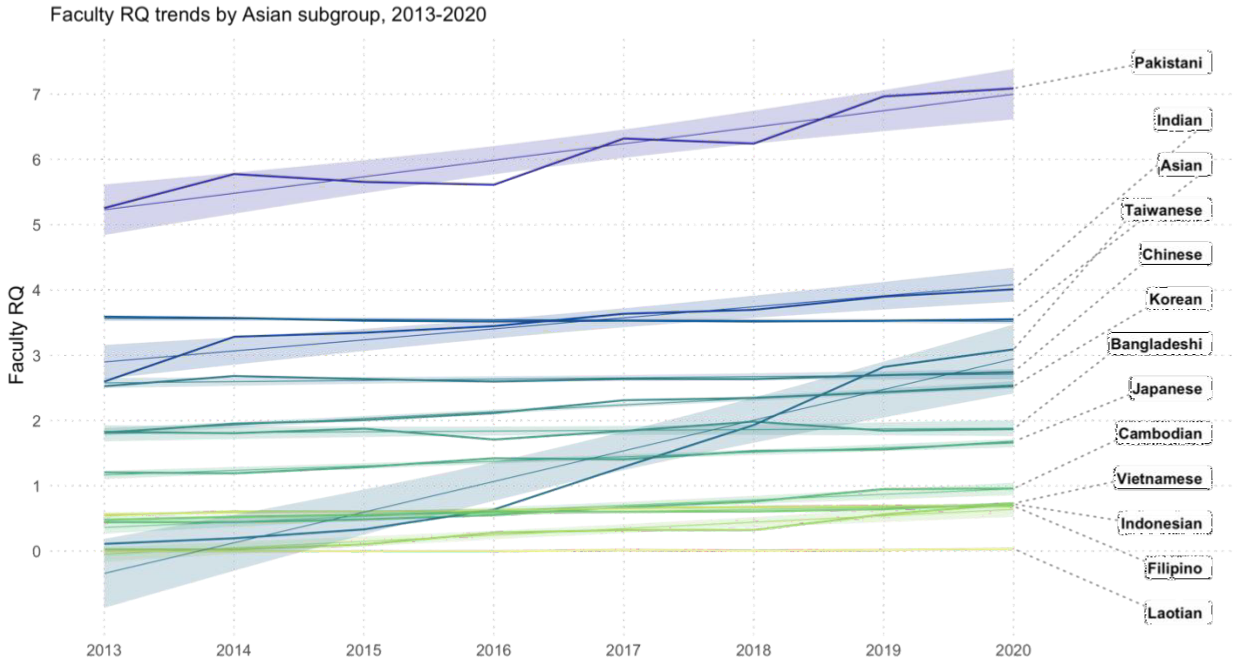

Abbreviation: RQ, Representation Quotient

© 2024 Santos PMG et al. *JAMA Network Open.*

eTable 1. Resident Representation by Specialty and Asian Subgroup, 2013-2021

		Cambodian		Filipino		Laotian	
	N	*n* (%)	Median (IQR) RQ	*n* (%)	Median (IQR) RQ	*n* (%)	Median (IQR) RQ
Anesthesiology	11,099	29 (0.3)	0.43 (0.26, 0.6)	504 (4.5)	0.74 (0.68, 0.79)	3 (0)	0.06 (0, 0.15)
Dermatology	2,322	6 (0.3)	0.36 (0, 0.61)	97 (4.2)	0.64 (0.51, 0.78)	—	—
Emergency Medicine	8,338	21 (0.3)	0.26 (0.2, 0.31)	497 (6)	0.62 (0.57, 0.67)	13 (0.2)	0.19 (0, 0.32)
Family Medicine	18,841	100 (0.5)	0.75 (0.7, 0.89)	1579 (8.4)	1.2 (1.13, 1.29)	39 (0.2)	0.36 (0.23, 0.44)
General Surgery	51,996	93 (0.2)	0.32 (0.28, 0.36)	2168 (4.2)	0.75 (0.73, 0.77)	19 (0)	0.08 (0.03, 0.11)
Internal Medicine	2,520	5 (0.2)	0.3 (0, 0.49)	84 (3.3)	0.52 (0.43, 0.59)	—	—
Neurology	4,732	5 (0.1)	0.17 (0, 0.3)	153 (3.2)	0.52 (0.44, 0.58)	3 (0.1)	0.14 (0, 0.37)
Neurosurgery	6,518	36 (0.6)	0.62 (0.46, 0.84)	552 (8.5)	0.93 (0.89, 0.97)	9 (0.1)	0.19 (0, 0.34)
OB/GYN	3,405	6 (0.2)	0.39 (0, 0.53)	96 (2.8)	0.62 (0.57, 0.66)	3 (0.1)	0.26 (0, 0.66)
Ophthalmology	4,264	0 (0)	—	184 (4.3)	0.41 (0.36, 0.45)	—	—
Orthopedic Surgery	2,899	0 (0)	—	152 (5.2)	0.87 (0.77, 0.98)	—	—
Otolaryngology	3,904	4 (0.1)	0.16 (0, 0.31)	162 (4.1)	0.63 (0.57, 0.72)	6 (0.2)	0.31 (0, 0.81)
Pathology	13,062	45 (0.3)	0.46 (0.38, 0.53)	970 (7.4)	0.97 (0.91, 1.02)	15 (0.1)	0.19 (0.1, 0.31)
Pediatrics	2,819	4 (0.1)	0.29 (0, 0.56)	189 (6.7)	1.28 (1.17, 1.38)	—	—
Plastic Surgery	1,332	0 (0)	—	31 (2.3)	0.34 (0.27, 0.43)	—	—
PM&R	10,633	43 (0.4)	0.62 (0.11, 1.07)	575 (5.4)	0.91 (0.87, 0.93)	12 (0.1)	0.24 (0.18, 0.3)
Psychiatry	1,805	0 (0)	—	25 (1.4)	0.31 (0.28, 0.33)	—	—
Radiation Oncology	9,477	20 (0.2)	0.41 (0.28, 0.64)	310 (3.3)	0.62 (0.57, 0.68)	—	—
Radiology	12,591	25 (0.2)	0.25 (0.18, 0.37)	722 (5.7)	0.74 (0.68, 0.78)	17 (0.1)	0.22 (0.18, 0.3)
Urology	2,470	2 (0.1)	0.1 (0, 0.1)	76 (3.1)	0.49 (0.45, 0.59)	4 (0.2)	0.34 (0, 0.67)

eTable 1. Resident Representation by Specialty and Asian Subgroup, 2013-2021 (continued)

		Bangladeshi		Chinese		Indian	
	N	*n* (%)	Median (IQR) RQ	*n* (%)	Median (IQR) RQ	*n* (%)	Median (IQR) RQ
Anesthesiology	11,099	120 (1.1)	3.25 (3.02, 3.74)	2763 (24.9)	3.41 (3.27, 3.59)	3520 (31.7)	3.78 (3.38, 4.14)
Dermatology	2,322	12 (0.5)	1.37 (1.06, 1.79)	665 (28.6)	3.6 (3.38, 3.71)	743 (32)	3.44 (3.36, 3.6)
Emergency Medicine	8,338	88 (1.1)	1.91 (1.1, 2.89)	2205 (26.4)	2.33 (2.18, 2.43)	2551 (30.6)	2.34 (2.18, 2.54)
Family Medicine	18,841	340 (1.8)	4.69 (4.5, 5.35)	3115 (16.5)	1.94 (1.75, 2.1)	6471 (34.3)	3.57 (3.19, 3.92)
General Surgery	51,996	1004 (1.9)	6.44 (6.05, 7.13)	9080 (17.5)	2.64 (2.38, 2.9)	22080 (42.5)	5.59 (4.82, 6.35)
Internal Medicine	2,520	9 (0.4)	0.98 (0, 1.63)	549 (21.8)	2.86 (2.75, 2.97)	961 (38.1)	4.4 (3.78, 5.1)
Neurology	4,732	94 (2)	5.93 (4.69, 6.93)	1102 (23.3)	3.18 (2.77, 3.51)	1767 (37.3)	4.38 (3.91, 4.78)
Neurosurgery	6,518	86 (1.3)	2.73 (2.36, 3.1)	1646 (25.3)	2.32 (2.27, 2.31)	2126 (32.6)	2.58 (2.53, 2.66)
OB/GYN	3,405	22 (0.6)	2.55 (0.98, 3.54)	937 (27.5)	5.01 (4.64, 5.32)	1163 (34.2)	5.48 (4.34, 6.26)
Ophthalmology	4,264	12 (0.3)	0.5 (0.36, 0.68)	1033 (24.2)	1.95 (1.86, 2.04)	1574 (36.9)	2.59 (2.23, 2.93)
Orthopedic Surgery	2,899	24 (0.8)	2.56 (2.24, 3.49)	784 (27)	3.73 (3.66, 3.79)	1020 (35.2)	4.22 (3.91, 4.44)
Otolaryngology	3,904	33 (0.8)	2.38 (1.28, 3.23)	1697 (43.5)	5.56 (4.8, 6.14)	720 (18.4)	2.07 (1.45, 2.55)
Pathology	13,062	211 (1.6)	3.91 (2.93, 4.84)	2532 (19.4)	2.13 (2.03, 2.22)	5076 (38.9)	3.7 (3.44, 3.97)
Pediatrics	2,819	19 (0.7)	2.32 (1.59, 3.48)	697 (24.7)	3.89 (3.75, 4.07)	959 (34)	4.68 (4.06, 5.23)
Plastic Surgery	1,332	9 (0.7)	1.57 (0, 3.07)	443 (33.3)	4.22 (4.07, 4.42)	381 (28.6)	3.26 (2.82, 3.69)
PM&R	10,633	230 (2.2)	6.66 (5.62, 8.1)	1875 (17.6)	2.42 (2.22, 2.51)	4067 (38.2)	4.72 (3.94, 5.43)
Psychiatry	1,805	18 (1)	4.06 (2.94, 5.81)	443 (24.5)	4.55 (4.38, 4.73)	759 (42)	6.79 (5.94, 7.5)
Radiation Oncology	9,477	69 (0.7)	2.6 (2.43, 2.81)	2016 (21.3)	3.38 (3.31, 3.44)	3764 (39.7)	5.52 (4.48, 6.17)
Radiology	12,591	118 (0.9)	2.23 (1.9, 2.62)	3284 (26.1)	2.79 (2.71, 2.85)	3659 (29.1)	2.72 (2.37, 3.05)
Urology	2,470	27 (1.1)	3.1 (2.16, 4.02)	765 (31)	3.98 (3.86, 4.14)	916 (37.1)	4.17 (3.95, 4.41)

© 2024 Santos PMG et al. *JAMA Network Open.*

eTable 1. Resident Representation by Specialty and Asian Subgroup, 2013-2021 (continued)

	Indonesian			Japanese		Korean	
	N	*n* (%)	Median (IQR) RQ	*n* (%)	Median (IQR) RQ	*n* (%)	Median (IQR) RQ
Anesthesiology	11,099	28 (0.3)	1.18 (0.96, 1.47)	308 (2.8)	1.49 (1.31, 1.62)	1220 (11)	3.52 (3.34, 3.68)
Dermatology	2,322	10 (0.4)	1.78 (1.12, 2.51)	73 (3.1)	1.51 (0.71, 2.38)	273 (11.8)	3.45 (3.3, 3.63)
Emergency Medicine	8,338	40 (0.5)	1.4 (1.1, 1.98)	425 (5.1)	1.77 (1.64, 1.87)	821 (9.8)	2.01 (1.87, 2.14)
Family Medicine	18,841	72 (0.4)	1.46 (0.64, 2.05)	685 (3.6)	1.68 (1.54, 1.92)	1297 (6.9)	1.91 (1.86, 1.98)
General Surgery	51,996	138 (0.3)	1.35 (1.07, 1.5)	1192 (2.3)	1.35 (1.24, 1.43)	3273 (6.3)	2.2 (2.15, 2.26)
Internal Medicine	2,520	1 (0)	0.18 (0, 0)	60 (2.4)	1.22 (1.04, 1.29)	326 (12.9)	3.96 (3.82, 4.12)
Neurology	4,732	21 (0.4)	1.98 (1.62, 2.57)	95 (2)	1.04 (0.75, 1.36)	340 (7.2)	2.24 (2.12, 2.39)
Neurosurgery	6,518	24 (0.4)	1.14 (0.67, 1.46)	359 (5.5)	1.98 (1.79, 2.19)	562 (8.6)	1.85 (1.78, 1.93)
OB/GYN	3,405	18 (0.5)	3.24 (2.5, 4.55)	99 (2.9)	2.08 (1.94, 2.23)	333 (9.8)	4.17 (3.96, 4.32)
Ophthalmology	4,264	20 (0.5)	1.28 (0.86, 1.68)	235 (5.5)	1.73 (1.65, 1.78)	495 (11.6)	2.18 (2.07, 2.28)
Orthopedic Surgery	2,899	6 (0.2)	0.97 (0.85, 1.33)	120 (4.1)	2.24 (1.87, 2.45)	289 (10)	3.23 (2.73, 3.67)
Otolaryngology	3,904	15 (0.4)	1.65 (0.95, 2.04)	187 (4.8)	2.38 (2.12, 2.62)	284 (7.3)	2.15 (1.77, 2.58)
Pathology	13,062	67 (0.5)	1.91 (1.46, 2.27)	455 (3.5)	1.5 (1.41, 1.61)	1033 (7.9)	2.03 (1.82, 2.28)
Pediatrics	2,819	14 (0.5)	2.71 (1.51, 3.47)	107 (3.8)	2.34 (1.88, 2.79)	255 (9)	3.3 (2.45, 4.14)
Plastic Surgery	1,332	9 (0.7)	2.67 (1.52, 3.27)	45 (3.4)	1.75 (1.43, 1.91)	166 (12.5)	3.62 (3.01, 4.32)
PM&R	10,633	37 (0.3)	1.58 (0.56, 2.45)	362 (3.4)	1.85 (1.65, 2.01)	921 (8.7)	2.8 (2.59, 3.12)
Psychiatry	1,805	6 (0.3)	2.09 (0, 3.48)	34 (1.9)	1.37 (1.2, 1.39)	178 (9.9)	4.28 (3.54, 5.05)
Radiation Oncology	9,477	25 (0.3)	1.46 (0.86, 1.94)	230 (2.4)	1.52 (1.33, 1.7)	914 (9.6)	3.6 (3.47, 3.83)
Radiology	12,591	41 (0.3)	1.17 (0.78, 1.47)	528 (4.2)	1.76 (1.66, 1.85)	1662 (13.2)	3.3 (3.25, 3.35)
Urology	2,470	16 (0.6)	2.72 (1.11, 4.05)	43 (1.7)	0.86 (0.52, 1.13)	197 (8)	2.4 (2.18, 2.69)

© 2024 Santos PMG et al. *JAMA Network Open.*

eTable 1. **Resident Representation by Specialty and Asian Subgroup, 2013-2021** (continued)

		Pakistani		Taiwanese		Vietnamese	
	N	*n* (%)	Median (IQR) RQ	*n* (%)	Median (IQR) RQ	*n* (%)	Median (IQR) RQ
Anesthesiology	11,099	587 (5.3)	6.5 (5.85, 7.26)	621 (5.6)	14.28 (12.39, 17.1)	986 (8.9)	2.84 (2.69, 3.09)
Dermatology	2,322	101 (4.3)	4.72 (4.47, 5.31)	136 (5.9)	13.07 (9.09, 17.69)	141 (6.1)	1.8 (1.63, 2.02)
Emergency Medicine	8,338	487 (5.8)	4.33 (3.73, 5.3)	523 (6.3)	10.41 (10.16, 11.51)	560 (6.7)	1.36 (1.17, 1.48)
Family Medicine	18,841	1788 (9.5)	10.06 (9.69, 10.48)	814 (4.3)	9.62 (9.15, 10.83)	1939 (10.3)	2.85 (2.58, 3.07)
General Surgery	51,996	5481 (10.5)	13.91 (13.85, 14.18)	2364 (4.5)	13.28 (11.87, 15.13)	2841 (5.5)	1.92 (1.71, 2.09)
Internal Medicine	2,520	191 (7.6)	8.76 (8.19, 9.17)	66 (2.6)	6.32 (5.27, 7.84)	90 (3.6)	1.11 (0.94, 1.26)
Neurology	4,732	481 (10.2)	12.2 (11.03, 13.31)	230 (4.9)	12.23 (9.56, 16.19)	197 (4.2)	1.31 (1.25, 1.39)
Neurosurgery	6,518	248 (3.8)	3.14 (2.88, 3.33)	357 (5.5)	9.64 (8.91, 10.23)	509 (7.8)	1.68 (1.39, 1.91)
OB/GYN	3,405	204 (6)	9.61 (8.86, 10.35)	193 (5.7)	19.08 (12.74, 25.91)	224 (6.6)	2.82 (2.51, 3.19)
Ophthalmology	4,264	256 (6)	4.16 (3.61, 4.76)	186 (4.4)	6.64 (6.17, 7.84)	158 (3.7)	0.69 (0.61, 0.78)
Orthopedic Surgery	2,899	104 (3.6)	4.34 (3.83, 4.8)	198 (6.8)	17.09 (11.13, 22.93)	141 (4.9)	1.58 (1.44, 1.77)
Otolaryngology	3,904	248 (6.4)	7.07 (6.13, 7.95)	183 (4.7)	11.34 (10.56, 13.21)	156 (4)	1.19 (1.1, 1.33)
Pathology	13,062	749 (5.7)	5.54 (5.34, 5.85)	680 (5.2)	10.98 (9.91, 12.37)	1013 (7.8)	1.99 (1.88, 2.09)
Pediatrics	2,819	164 (5.8)	8.15 (7.46, 8.73)	129 (4.6)	13.46 (12.91, 16.16)	223 (7.9)	2.92 (2.63, 3.22)
Plastic Surgery	1,332	41 (3.1)	3.58 (3.28, 3.83)	97 (7.3)	16.56 (14.01, 20.28)	89 (6.7)	2.04 (1.84, 2.39)
PM&R	10,633	1083 (10.2)	12.91 (11.2, 13.99)	416 (3.9)	9.79 (6.5, 12.58)	638 (6)	1.94 (1.59, 2.34)
Psychiatry	1,805	98 (5.4)	9.05 (7.83, 10.42)	98 (5.4)	18.56 (13.69, 27.26)	68 (3.8)	1.64 (1.5, 1.75)
Radiation Oncology	9,477	627 (6.6)	9.33 (8.56, 10)	442 (4.7)	13.98 (9.52, 18.37)	560 (5.9)	2.22 (1.91, 2.58)
Radiology	12,591	598 (4.7)	4.53 (4.19, 4.83)	719 (5.7)	11.49 (10.62, 12.66)	856 (6.8)	1.7 (1.61, 1.78)
Urology	2,470	33 (1.3)	1.53 (0.97, 2.05)	172 (7)	16.29 (12.24, 21.83)	128 (5.2)	1.56 (1.34, 1.92)

© 2024 Santos PMG et al. *JAMA Network Open.*

eTable 2. Resident RQ Trends by Specialty and Asian Subgroup, 2013 to 2021

	Cambodian		Filipino		Laotian	
	RQ Slope (95% CI)	*p*-value	RQ Slope (95% CI)	*p*-value	RQ Slope (95% CI)	*p*-value
Anesthesiology	0.06 (0.04, 0.09)	<0.001	0.02 (0.007, 0.04)	0.01	-0.005 (-0.04, 0.03)	0.68
Dermatology	0.15 (0.09, 0.21)	<0.001	-0.04 (-0.12, 0.03)	0.22	—	—
Emergency Medicine	0.03 (0.009, 0.06)	0.02	0.03 (0.02, 0.04)	<0.001	0.09 (0.05, 0.13)	0.003
ENT	—	—	-0.01 (-0.07, 0.05)	0.69	—	—
Family Medicine	0.07 (-0.002, 0.14)	0.06	-0.04 (-0.06, -0.01)	0.01	0.08 (0.03, 0.13)	0.009
General Surgery	0.03 (-5.94, 0.06)	0.09	-0.02 (-0.04, 0.008)	0.14	-0.03 (-0.05, -0.003)	0.03
Internal Medicine	0.02 (-0.009, 0.04)	0.16	0.002 (-0.004, 0.009)	0.37	0.02 (0.001, 0.04)	0.04
Neurology	-0.002 (-0.06, 0.06)	0.93	-0.0004 (-0.04, 0.04)	0.98	-0.04 (-0.11, 0.03)	0.19
Neurosurgery	0.08 (0.01, 0.14)	0.02	0.33 (0.02, 0.05)	0.004	—	—
OB/GYN	-0.02 (-0.13, 0.08)	0.59	0.1 (0.004, 0.02)	0.01	0.09 (0.05, 0.14)	0.003
Ophthalmology	0.03 (-0.11, 0.18)	0.58	-0.004 (-0.03, 0.02)	0.72	0.12 (0.05, 0.19)	0.007
Orthopedic Surgery	—	—	0.02 (0.01, 0.03)	0.001	—	—
Pathology	0.06 (0.02, 0.09)	0.006	0.02 (-0.01, 0.06)	0.19	0.15 (0.06, 0.24)	0.008
Pediatrics	0.01 (-0.03, 0.05)	0.45	0.01 (-0.01, 0.03)	0.2	-0.01 (-0.05, 0.03)	0.43
Plastic Surgery	—	—	0.04 (0.02, 0.07)	0.008	—	—
PM&R	-0.06 (-0.17, 0.06)	0.26	-0.05 (-0.15, 0.05)	0.24	—	—
Psychiatry	0.23 (0.13, 0.32)	<0.001	-0.005 (-0.02, 0.01)	0.55	0.02 (0.005, 0.04)	0.02
Radiation Oncology	—	—	-0.008 (-0.04, 0.02)	0.53	—	—
Radiology	0.09 (0.06, 0.13)	<0.001	-0.03 (-0.05, -0.01)	0.01	—	—
Urology	0.06 (7.90, 0.11)	0.03	-0.04 (-0.07, -0.02)	0.006	-0.07 (-0.20, 0.07)	0.26

	Bangladeshi		Chinese		Indian	
	RQ Slope (95% CI)	*p*-value	RQ Slope (95% CI)	*p*-value	RQ Slope (95% CI)	*p*-value
Anesthesiology	0.33 (0.06, 0.60)	0.02	-0.10 (-0.15, -0.05)	0.004	-0.20 (-0.27, -0.13)	<0.001
Dermatology	0.24 (0.09, 0.39)	0.008	-0.12 (-0.21, -0.02)	0.02	-0.03 (-0.13, 0.06)	0.41
Emergency Medicine	0.40 (0.30, 050)	<0.001	-0.06 (-0.11, -0.01)	0.02	-0.10 (-0.15, -0.05)	0.003
ENT	0.08 (-0.41, 0.56)	0.71	-0.02 (-0.07, 0.03)	0.28	-0.12 (-0.20, -0.04)	0.01
Family Medicine	0.31 (0.07, 0.55)	0.02	0.04 (-0.01, 0.09)	0.13	-0.19 (-0.26, -0.12)	<0.001
General Surgery	0.14 (-7.96, 0.28)	0.06	-0.06 (-0.11, -0.009)	0.03	-0.16 (-0.24, 0.08)	0.003
Internal Medicine	0.22 (-0.04, 0.48)	0.09	-0.12 (-0.16, -0.08)	<0.001	-0.35 (-0.46, -0.24)	<0.001
Neurology	0.25 (-0.43, 0.93)	0.40	-0.21 (-0.31, -0.11)	0.003	-0.025 (-0.38, -0.12)	0.003
Neurosurgery	0.29 (0.13, 0.46)	0.005	-0.07 (-0.12, -0.03)	0.006	-0.30 (0.44, -0.16)	0.002
OB/GYN	-0.01 (-0.25, 0.23)	0.92	-0.02 (-0.06, 0.02)	0.22	-0.03 (-0.09, 0.04)	0.33
Ophthalmology	0.78 (0.39, 1.17)	0.003	-0.0009 (-0.26, 0.16)	0.99	-0.49 (-0.65, 0.33)	<0.001
Orthopedic Surgery	0.04 (-0.06, 0.14)	0.37	-0.06 (-0.08, -0.04)	<0.001	-0.16 (-0.23, -0.08)	0.002
Pathology	0.45 (0.24, 0.66)	0.002	-0.43 (-0.48, -0.37)	<0.001	-0.25 (-0.33, -0.17)	<0.001
Pediatrics	0.43 (0.16, 0.71)	0.008	-0.06 (-0.09, -0.02)	0.007	-0.15 (-0.23, -0.08)	0.003
Plastic Surgery	0.61 (0.29, 0.94)	0.004	0.01 (-0.11, 0.12)	0.84	-0.22 (-0.33, -0.10)	0.003
PM&R	0.30 (-0.18, 0.77)	0.18	-0.04 (-0.13, 0.05)	0.33	-0.26 (-0.35, -0.17)	<0.001
Psychiatry	0.49 (-0.22, 1.21)	0.14	0.08 (0.03, 0.13)	0.006	-0.37 (-0.48, -0.25)	<0.001
Radiation Oncology	0.57 (-0.17, 1.32)	0.11	-0.04 (-0.15, 0.06)	0.34	-0.41 (-0.62, -0.20)	0.003
Radiology	0.03 (-0.15, 0.20)	0.73	-0.02 (-0.07, 0.03)	0.34	-0.49 (-0.61, -0.37)	<0.001
Urology	0.30 (-0.09, 0.69)	0.11	0.03 (-0.03, 0.08)	0.31	-0.13 (-0.26, 0.004)	0.06

* Significance set at *P*=0.0025 using the Bonferroni correction method for multiple comparisons.

© 2024 Santos PMG et al. *JAMA Network Open.*

eTable 2. Resident RQ Trends by Specialty and Asian Subgroup, 2013 to 2021* (continued)

	Indonesian		Japanese		Korean	
	RQ Slope (95% CI)	*p*-value	RQ Slope (95% CI)	*p*-value	RQ Slope (95% CI)	*p*-value
Anesthesiology	0.12 (-0.05, 0.31)	0.14	0.04 (-0.04, 0.12)	0.23	-0.03 (-0.13, 0.07)	0.50
Dermatology	0.42 (0.05, 0.80)	0.03	0.29 (0.12, 0.47)	0.006	-0.10 (-0.19, -0.01)	0.03
Emergency Medicine	0.20 (-0.03, 0.43)	0.07	-0.04 (-0.10, 0.1)	0.10	0.04 (-0.005, 0.09)	0.07
ENT	0.17 (-0.002, 0.35)	0.05	0.15 (-1.00, 0.30)	0.05	-0.24 (-0.31, -0.17)	<0.001
Family Medicine	0.38 (0.32, 0.43)	<0.001	0.11 (0.05, 0.17)	0.005	0.01 (-0.02, 0.05)	0.37
General Surgery	0.20 (0.07, 0.34)	0.01	-0.02 (-0.08, 0.04)	0.51	-0.02 (-0.06, 0.01)	0.15
Internal Medicine	0.09 (-0.02, 0.20)	0.09	0.009 (-0.05, 0.06)	0.69	0.03 (-0.03, 0.10)	0.28
Neurology	0.16 (-0.02, 0.34)	0.07	0.07 (-0.02, 0.17)	0.10	0.04 (-0.04, 0.12)	0.29
Neurosurgery	0.11 (-0.05, 0.28)	0.13	0.09 (0.01, 0.16)	0.03	0.09 (-0.03, 0.20)	0.11
OB/GYN	0.26 (0.19, 0.33)	<0.001	0.08 (0.003, 0.15)	0.04	0.02 (-0.01, 0.06)	0.13
Ophthalmology	0.29 (-0.37, 0.95)	0.32	0.01 (-0.09, 0.11)	0.82	-0.15 (-0.26, -0.04)	0.02
Orthopedic Surgery	-0.03 (-0.29, 0.23)	0.79	-0.01 (-0.06, 0.04)	0.55	-0.07 (-0.13, -0.02)	0.02
Pathology	0.18 (-0.04, 0.40)	0.10	-0.02 (-0.15, 0.12)	0.78	-0.19 (-0.26, -0.12)	<0.001
Pediatrics	0.32 (0.23, 0.41)	<0.001	0.05 (7.51, 0.10)	0.05	-0.03 (-0.14, 0.08)	0.53
Plastic Surgery	0.77 (0.32, 1.22)	0.006	-0.09 (-0.25, 0.06)	0.19	-0.28 (0.13, 0.43)	0.004
PM&R	0.07 (-0.82, 0.96)	0.85	0.08 (-0.15, 0.31)	0.41	0.29 (-0.003, 0.59)	0.05
Psychiatry	0.40 (0.15, 0.65)	0.008	0.11 (0.05, 0.17)	0.004	0.15 (0.05, 0.24)	0.008
Radiation Oncology	0.94 (0.53, 1.35)	0.001	0.06 (-0.05, 0.17)	0.21	-0.31 (-0.49, -0.14)	0.005
Radiology	0.36 (0.19, 0.53)	0.002	0.05 (-0.02, 0.12)	0.15	0.12 (-0.02, 0.26)	0.08
Urology	0.71 (0.32, 1.10)	0.004	0.14 (0.03, 0.26)	0.02	0.11 (0.03, 0.19)	0.02
	**Pakistani**		**Taiwanese**		**Vietnamese**	
	RQ Slope (95% CI)	*p*-value	RQ Slope (95% CI)	*p*-value	RQ Slope (95% CI)	*p*-value
Anesthesiology	-0.37 (-0.61, -0.13)	0.01	1.60 (0.61, 2.58)	0.008	0.10 (-0.002, 0.19)	0.05
Dermatology	0.20 (-0.24, 0.65)	0.31	2.61 (1.52, 3.70)	0.001	-0.09 (-0.16, -0.02)	0.02
Emergency Medicine	0.33 (0.11, 0.55)	0.009	0.28 (-0.51, 1.08)	0.42	0.11 (0.08, 0.15)	<0.001
ENT	0.15 (-0.06, 0.36)	0.14	3.61 (2.64, 4.58)	<0.001	-0.03 (-0.13, 0.06)	0.44
Family Medicine	-0.32 (-0.52, -0.11)	0.009	0.52 (-0.11, 1.16)	0.09	0.14 (0.08, 0.20)	0.001
General Surgery	-0.16 (-0.27, -0.05)	0.01	0.76 (0.36, 1.16)	0.003	0.04 (0.01, 0.07)	0.02
Internal Medicine	0.03 (-0.14, 0.19)	0.70	-0.39 (-1.11, 0.34)	0.24	0.07 (0.02, 0.11)	0.02
Neurology	-0.50 (-1.02, 0.03)	0.06	0.71 (-1.40, 2.81)	0.44	-0.005 (-0.08, 0.07)	0.87
Neurosurgery	0.06 (-0.31, 0.43)	0.71	0.91 (0.38, 1.43)	0.006	-0.08 (-0.16, -0.007)	0.04
OB/GYN	-0.17 (-0.35, -0.001)	0.05	0.18 (-0.27, 0.63)	0.37	0.12 (0.07, 0.17)	<0.001
Ophthalmology	0.33 (-0.03, 0.68)	0.06	4.47 (3.41, 5.53)	<0.001	0.11 (-0.04, 0.26)	0.14
Orthopedic Surgery	0.25 (0.06, 0.43)	0.02	0.26 (-0.42, 0.94)	0.39	0.03 (-0.01, 0.06)	0.14
Pathology	0.42 (0.07, 0.77)	0.03	0.63 (-0.04, 1.29)	0.06	0.0008 (-0.05, 0.06)	0.97
Pediatrics	0.08 (-0.08, 0.25)	0.27	0.17 (-0.87, 1.20)	0.71	0.08 (0.03, 0.12)	0.006
Plastic Surgery	-0.16 (-0.43, -0.11)	0.19	2.08 (1.51, 2.65)	<0.001	0.03 (-0.15, 0.20)	0.73
PM&R	-0.17 (-0.42, 0.09)	0.16	1.25 (0.06, 2.45)	0.04	0.15 (0.02, 0.27)	0.03
Psychiatry	-0.99 (-1.30, -0.68)	<0.001	1.62 (1.19, 2.05)	<0.001	0.17 (0.08, 0.25)	0.003
Radiation Oncology	-0.60 (-1.19, -0.006)	0.05	3.26 (-4.32, 6.57)	0.05	0.09 (0.010, 0.18)	0.03
Radiology	0.26 (-0.006, 0.52)	0.05	2.55 (1.93, 3.17)	<0.001	0.16 (0.13, 0.19)	<0.001
Urology	-0.09 (-0.40, 0.22)	0.50	2.57 (0.68, 4.45)	0.02	0.13 (-0.002, 0.26)	0.05

* Significance set at *P*=0.0025 using the Bonferroni correction method for multiple comparisons.

© 2024 Santos PMG et al. *JAMA Network Open.*
