# Supplementary material for: Asian American Representation in Medicine by Career Stage and Residency Specialty
Source: JAMA Netw Open. 2024 Nov 19;7(11):e2444478. doi: 10.1001/jamanetworkopen.2024.44478 (PMC11577148; doi:10.1001/jamanetworkopen.2024.44478)
Supplement: Supplement 2. — Data Sharing Statement [file jamanetwopen-e2444478-s002.pdf]

Data Sharing Statement

Data
Data available: Yes
Data types: Deidentified participant data
How to access data: https://www.aamc.org/request-aamc-data santosp@mskcc.org
When available: With publication

Supporting Documents
Document types: None

Additional Information
Who can access the data: researchers whose proposed use of the data has been approved
Types of analyses: for a specified purpose
Mechanisms of data availability: after approval of a proposal
